# Supplementary material for: Impact of HIV-Related Immune Impairment of Yellow Fever Vaccine Immunogenicity in People Living with HIV—ANRS 12403
Source: Vaccines (Basel). 2024 May 25;12(6):578. doi: 10.3390/vaccines12060578 (PMC11209244; doi:10.3390/vaccines12060578)
Supplement: Supplementary file 1 [file vaccines-12-00578-s001.zip › vaccines-2986640-supplementary.pdf]

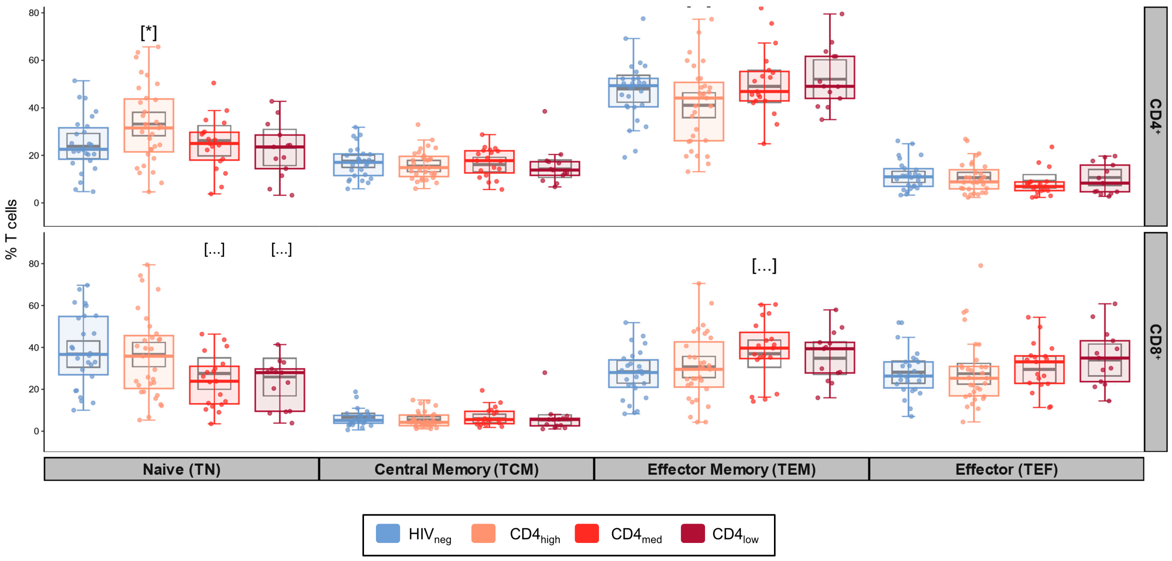

Figure S1. Frequencies of T cell subsets in PLWH and control groups. … $p < 0.1$; * $p < 0.05$.

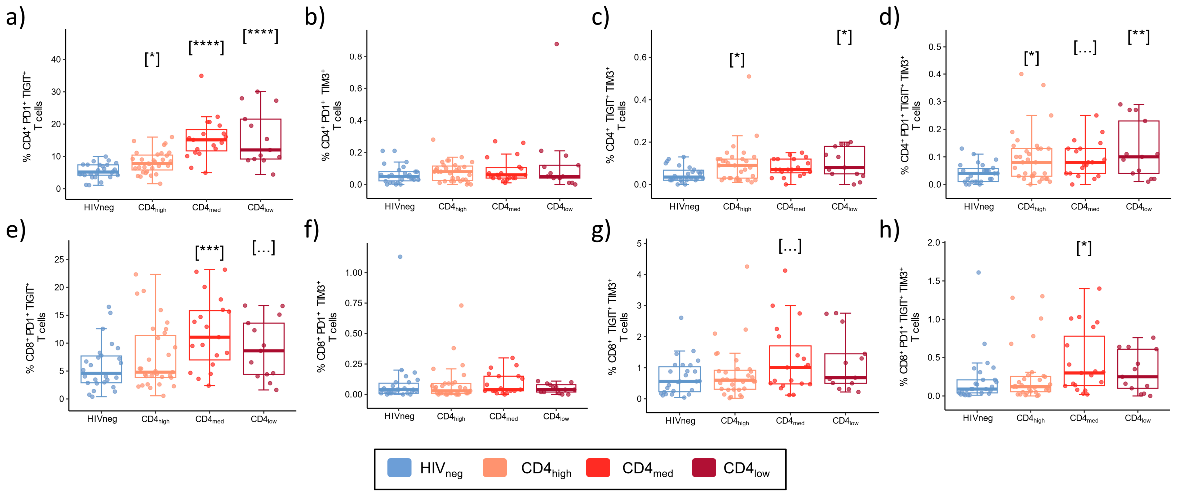

Figure S2. Boolean combination of exhaustion markers in total T cells in PLWH and control groups. … $p < 0.1$; * $p < 0.05$; ** $p < 0.01$; *** $p < 0.001$; and **** $p < 0.0001$.

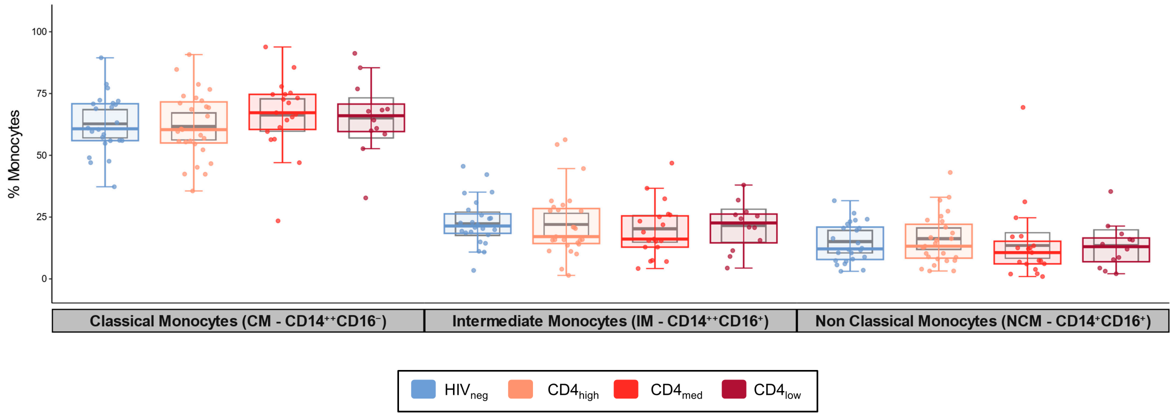

Figure S3. Monocyte subsets in PLWH and control groups.

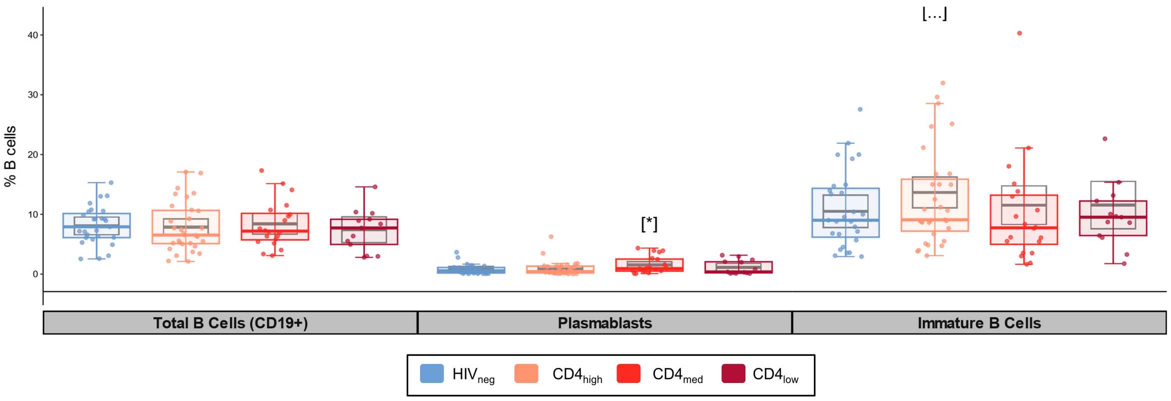

Figure S4. Total B cells, plasmablasts, and immature B cells in PLWH and control groups. … $p < 0.1$; * $p < 0.05$.

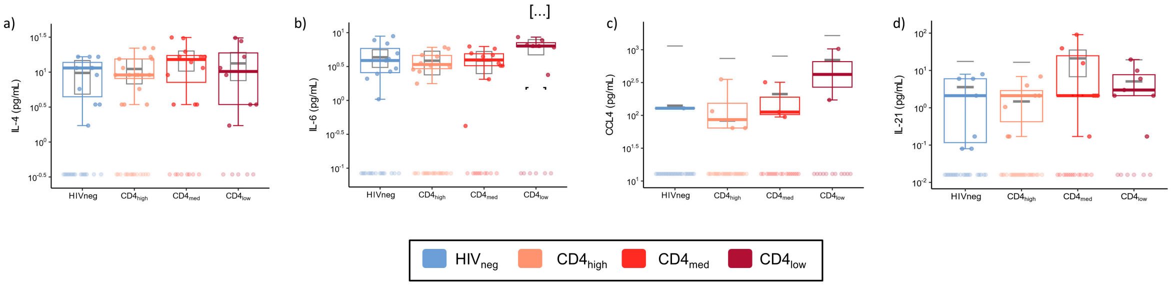

Figure S5. Plasma levels of soluble markers in PLWH and control groups. … $p < 0.1$.
